# Lung cancer stem cells and their aggressive progeny, controlled by EGFR/MIG6 inverse expression, dictate a novel NSCLC treatment approach

**DOI:** 10.18632/oncotarget.26817

**Published:** 2019-04-02

**Authors:** Zhiguang Xiao, Bianca Sperl, Silvia Gärtner, Tatiana Nedelko, Elvira Stacher-Priehse, Axel Ullrich, Pjotr G. Knyazev

**Affiliations:** ^1^ Department of Molecular Biology, Max-Planck Institute of Biochemistry, Martinsried, Munich, 82152, Germany; ^2^ Department of Medicine, University of Rochester School of Medicine and Dentistry, Rochester, NY 14642, USA; ^3^ Department of Medicine III, Klinikum rechts der Isar, TUM, Munich, 81675, Germany; ^4^ Asclepius Institute of Pathology, Gauting, 82131, Germany; ^5^ Current address: DoNatur GmbH, Martinsried, Munich, 82152, Germany

**Keywords:** NSCLC, cancer stem cells, niches, EGFR/MIG6, drug resistance

## Abstract

The lung cancer stem cell (LuCSC) model comprises an attractive framework to explore acquired drug resistance in non-small cell lung cancer (NSCLC) treatment. Here, we used NSCLC cell line model to translate cellular heterogeneity into tractable populations to understand the origin of lung cancers and drug resistance. The epithelial LuCSCs, presumably arising from alveolar bipotent stem/progenitor cells, were lineage naïve, noninvasive, and prone to creating aggressive progeny expressing AT2/AT1 markers. LuCSC-holoclones were able to initiate rimmed niches, where their specialization created pseudo-alveoli structures. Mechanistically, LuCSC transitioning from self-renewal (β-catenin and Nanog signaling) to malignant lineage differentiation is regulated by EGFR activation and the inverse inhibition of tumor suppressor MIG6. We further identified the functional roles of endogenous EGFR signaling in mediating progeny invasiveness and their ligands in LuCSC differentiation. Importantly, drug screening demonstrated that EGFR driving progeny were strongly responsive to TKIs; however, the LuCSCs were exclusively resistant but sensitive to AMPK agonist Metformin, antibiotic Salinomycin and to a lesser degree Carboplatin. Our data reveals previously an unknown mechanism of NSCLC resistance to EGFR-TKIs, which is associated with LuCSCs bearing a silenced EGFR and inversely expressed MIG6 suppressor gene. Taken altogether, successful NSCLC treatment requires development of a novel combination of drugs, efficiently targeting both LuCSCs and heterogeneous progeny.

## INTRODUCTION

Metastatic non-small cell lung cancer (NSCLC) cannot be cured with systemic chemotherapy and only 4% have a 5 year survival rate after postoperative (adjuvant Cisplatin-based) chemotherapy [1]. It has long been postulated that intratumoral heterogeneity contributes to disease progression, impacts therapeutic efficacy and therefore affects patient survival. The tumor hierarchical model suggests that cancer stem cells (CSCs), which represent a biologically distinct subset within the total cancer cell population, have the principal properties of self-renewal, clonal tumor initiation capacity and clonal long-term repopulation potential [2]. Analysis of dermal stem cells suggested that the tissue stem cell and the niche-forming cell are a single entity [3]. The rimmed clone formation was also recognized as a cell-intrinsic property [4]. However, distinct origins of the niche and stem cell have been suggested as well [5]. Up to now, the role of the niche in the regulation of lung CSCs (LuCSCs), adenocarcinoma development and metastatic progression is still a matter of debate and speculation.

A long-standing hypothesis proposes that abnormal EGFR signaling could abrogate harmonically regulated normal alveoli formation, leading to putative lung stem cell transformation into LuCSC traits. The deregulations involve hijacking stem cell self-renewal and its multi-lineage differentiation ability. Subsequently, activation of oncogenes leads to adenocarcinoma development [6–9]. Patients whose tumors are driven by EGFR classical mutations have been shown to respond well to EGFR tyrosine kinase inhibitors (EGFR-TKIs), including Gefitinib, Erlotinib, Afatinib and Osimertinib [10–13]. Nevertheless, targeting these mutations with kinase inhibitors is not curative in advanced disease. Several mechanisms have been proposed for patients` acquired drug resistance, but many mechanisms still remain, as well as the role of LuCSCs.

Genomic studies demonstrate that tumor suppressors were frequently mutated in NSCLCs. One of them is MIG6 (the product of mitogen-inducible gene 6, also known as ERRFI1), whose inactivation contributes to hyper-activation of the EGFR-module and MET signaling pathway [14, 15]. We and others have shown that genomic disruption of the MIG6 gene orchestrates constitutive EGFR signaling resulting in degenerative joint diseases, skin hyperplasia, melanomas, and lung cancer in mice [16, 17]. In human lung adenocarcinomas, MIG6 was associated with EGFR-TKI resistance of putative dormant cancer cells and epithelial mesenchymal transition (EMT) induced refractoriness [18].

To address some issues mentioned above, we isolated LuCSC-holoclones and their progeny from NSCLC cell lines to gain insight into the transcriptional machinery and signaling pathway controlling LuCSC self-renewal, niche formation and differentiation. We demonstrated the importance of the MIG6/EGFR modules in regulating cross-talk between diverse cell populations and also screened for potential drugs that are able to eradicate LuCSCs and their progeny.

## RESULTS

### Lung cancer cell lines exhibit cellular heterogeneity with distinct clonal morphologies and unique characteristics

**Figure 1:**
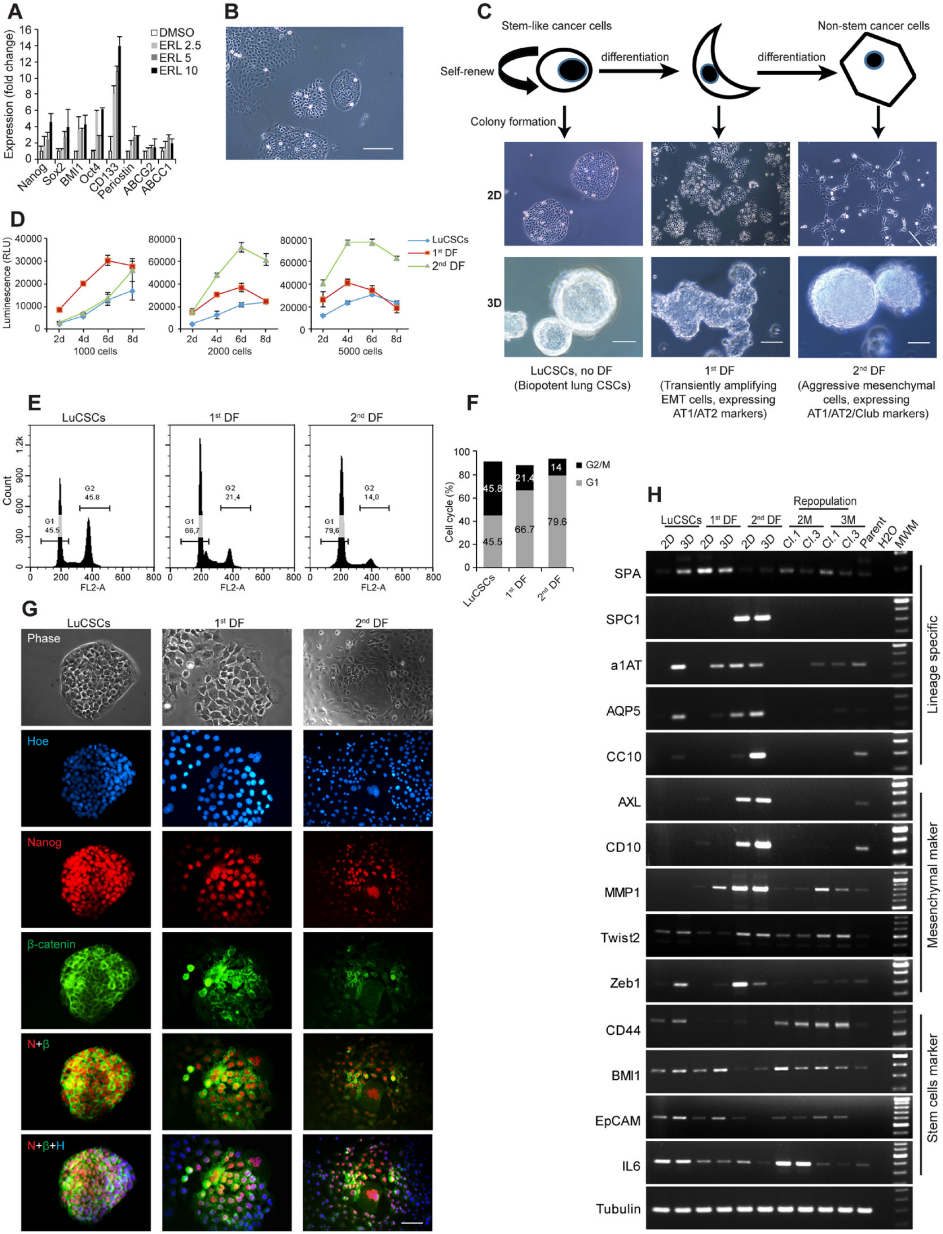
NSCLC cell line model (NCI-H1568) exhibits unique cellular heterogeneity and functional characteristic **(A)** Real-time PCR evaluation of stem cell markers on NCI-H1568 parent cells treated with various concentrations of Erlotinib as indicated. **(B)** Representative image of NCI-H1568 parent cells. Scale bar: 10 μm. **(C)** Schematic representation of hierarchical organization in NSCLC cell lines. Three major cell populations and their functional definition are indicated. Bright field of monolayer cells (2D) and alveospheres (3D) generated from distinct subclones from NCI-H1568 cell line. Scale bar: 10 μm for 2D, 20 μm for 3D. **(D)** Long-term (up to 8 days) proliferation status of distinct types of cell clones. 1000, 2000 and 5000 cells of each clone were seeded in 96-well plates, every other day the number of viable cells was evaluated using CellTiter-Glo cell viability assay. **(E)** Cell cycle analysis was performed after propidium iodide DNA labeling and subsequent flow cytometry. **(F)** Percentage of LuCSCs, 1^st^ DF and 2^nd^ DF cells in G1, S and G2-M phases of the cell cycle. **(G)** Immunofluorescence analysis of LuCSCs, 1^st^ DF and 2^nd^ DF cells with primary antibodies against Nanog (red) and ß-catenin (green) and the nuclear stain Hoechst 33342 (blue). Scale bar: 20 μm. **(H)** Gene expression profiles of 2D and 3D cells from NCI-H1568 distinct subclones, as well as LuCSC derived alveospheres after spontaneous long-term differentiation program. Conventional PCR was used for evaluation. M: months. Cl: clone.

Phenotypic heterogeneity is a common feature of NSCLC cell lines and attributes to genetic instability and clonal evolution of tumors. Cellular polymorphism is remarkable in EGFR wild type NCI-H1568 cells, which is a cell line derived from lymph node metastasis of lung adenocarcinoma. For cells after Erlotinib treatment, real-time PCR demonstrated the elevated expression of stem cell markers-Nanog, Sox2, BMI1, Oct4, CD133, Periostin, ABCG2 and ABCC1 (Figure 1A), suggesting that a cell population with low- or no expression of EGFR is resistant to Erlotinib, and their survival displays a high degree of mRNA-transcripts associated with stem cell and iPSC phenotypes [19]. To better characterize the cellular heterogeneity of NCI-H1568 cells (Figure 1B), cell sorting and monoclonal cultivation was carried out to determine the diversity of clonal morphologies. Three-major hierarchical organized cell populations are schematically shown in Figure 1C. Holoclones were homogeneous, tightly packed epithelial cobblestone-like cells with near circular borderlines. Since holoclones correspond closely to stem cells [20], we could conclude that they are in fact LuCSCs [21]. 1^st^ differentiated (DF) cells, an intermediate stage of differentiation, were considered as temporarily amplified mesenchymal cells. 2^nd^ DF cells are classified as the poorly differentiated epithelial AT1- and cuboidal AT2- malignant pneumocytes in terms of lineage specific marker expressions (Figure 1H). Similar experiments were carried out on cells derived from another six human NSCLC cell lines (NCI-H1975, NCI-H1650, NCI-H3122, HCC95, HCC827 and A549), one immortalized human small airway epithelial cell line (SALE) and one SALER cell line (SALE transformed by KRAS^G12V^ mutant form). All cell lines developed a range of colony morphologies resembling LuCSCs, 1^st^ DF cells and 2^nd^ DF cells of NCI-H1568 (Supplementary Figure 1A). All these clones from NCI-H1568 cells have the ability to form spheres (Figure 1C). LuCSC-holoclones spontaneously generated acini-like structures. These type of spheres could be produced by epithelial normal and cancer cell lines [22, 23]. 1^st^ DF cells were loosely connected initially but gradually formed compact alveospheres, partially resembling acini-like structures. In contrast, 2^nd^ DF cells formed tightly packed spheroids with an initial cell number of ~50, and quickly gathered more cells layer by layer, making the spheres denser without apoptotic cells inside.

Proliferation capability assay indicated that the stem-like cells are in a more quiescent state, which makes them theoretically less sensitive to chemotherapeutics that target rapidly dividing cells (Figure 1D). Further evaluation was performed by using the Ki67 index (Supplementary Figure 1B). In conjunction with the proliferation assay, FACS analysis revealed significant cell cycle replication state differences between LuCSCs, 1^st^ and 2^nd^ DF cells (Figure 1E). The proportion of cells in the G2-M phase of the cell cycle was approximately 2 and 3 folds higher in LuCSCs than in the 1^st^ and 2^nd^ DF cells, with sequential percentages around 46%, 21%, and 14% (Figure 1F). Apoptotic cells were virtually undetectable in all cases. Stem-like cells were considered to have a resting or a slow mitotic index, while they continued to replicate through extending G2 phase and repairing DNA damage caused by the exogenous stimuli. Immunofluorescent staining further demonstrated that LuCSC colonies were close to 100% positive for Nanog nuclear and ß-catenin cell periphery staining. The majority of 2^nd^ DF cells had low expression of Nanog, and only a few distinct ß-catenin positive cells were detected in this population. Both antibodies displayed moderate staining in 1^st^ DF cells (Figure 1G and Supplementary Figure 1C).

To seek for the expression of lineage-specific and other critical genes, we established three monoclonal cell lines and their alveospheres as well as two LuCSC monolayer clones for repopulation program (Figure 1H). 2D LuCSCs do not express either lineage-specific mRNAs, including the AT1 cell marker aquaporin 5 (AQP5), AT2 marker surfactant protein A, C (SPA, SPC1), alpha 1 antitrypsin (α1AT), and club cell secretory protein (CC10). However, all these transcripts except SPC1 and CC10 were pronounced in LuCSC alveospheres. In addition, 2D holoclones expressed CD44, BMI1, EpCAM and IL6 which were frequently reported to function as stem cell markers, but not the mesenchymal markers AXL, CD10, MMP1, and Zeb1. Although Twist2 was detectable in LuCSCs, the expression was strongly increased upon differentiation in the alveospheres. However, transcription factor-Zeb1, a real player of EMT, is still silent. Among 2D cell comparisons, the lineage-specific and mesenchymal markers were all highly expressed in 2^nd^ DF cells and consistently expressed under 3D culture conditions. Overall, 1^st^ DF cells, as a transiently amplifying population, expressed some stem cell markers and exhibited AT2 transition into AT1 phenotype. Single LuCSC spheres, after a 2- and 3-month differentiation, continuously expressed the main lineage and stem cell markers; however, SPC1, AXL and CD10 were hardly detectable in the repopulated cells at this early time point. All analyzed molecules, except EpCAM, were expressed in the parent cells. Value changes have been analyzed and quantified in histograms (Supplementary Figure 1D).

### LuCSCs in the rimmed-like niche formed pseudo-alveoli, which orchestrates differentiation into AT2/AT1 cell lineages

**Figure 2:**
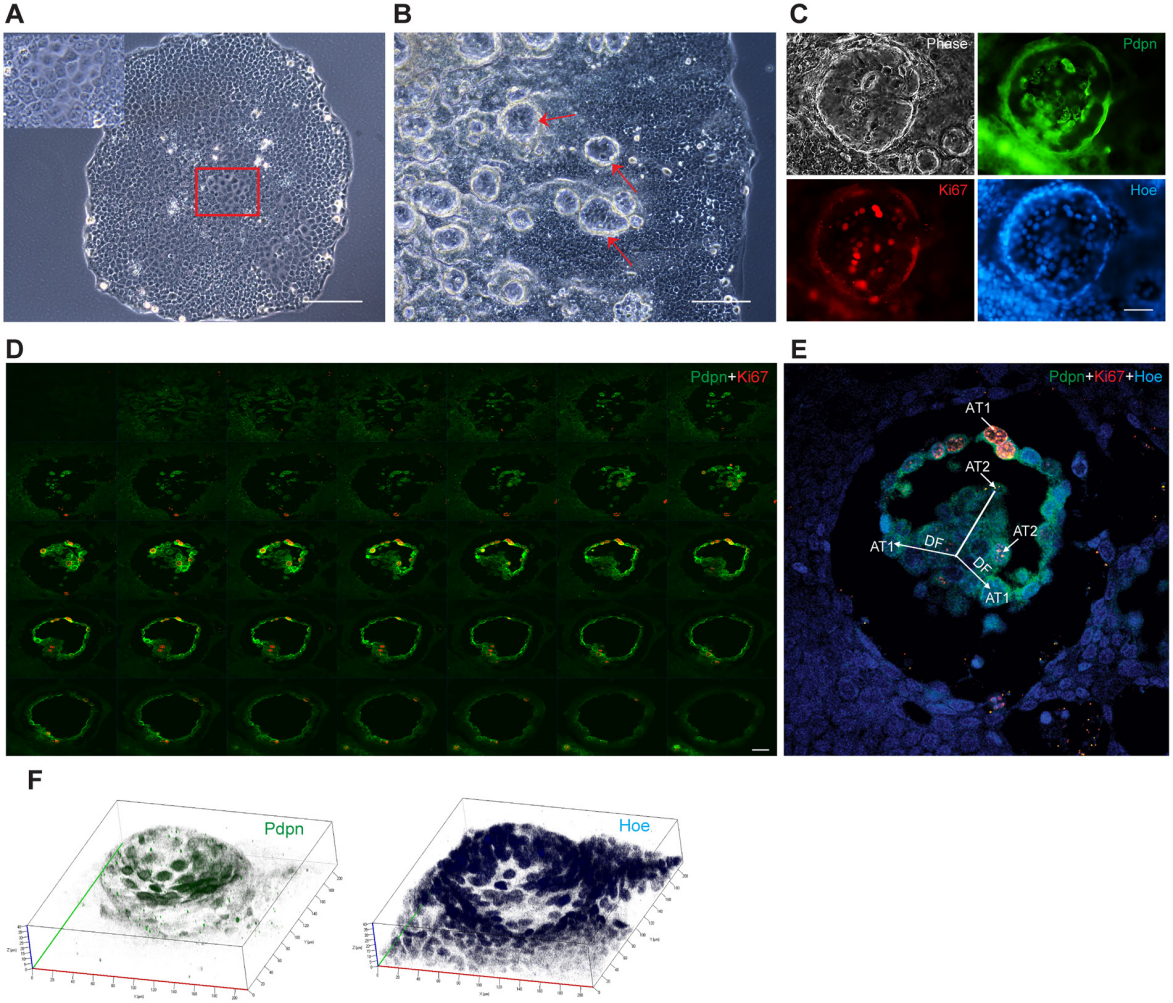
LuCSC-holoclones initiate rim-like niches and their differntiation creates pseudo-alveoli structures **(A)** Representative image of a LuCSC holoclone after 96 hrs of growth. Framed area indicates the putative initiation of niche formation where locally spindle-like cells appear. **(B)** Multiple rimmed-niche formation (red arrow) in a LuCSC holoclone after 84 days of growth and differentiation. **(C)** Immunofluorescence analysis of single niche with AT1 cell marker Pdpn (green), proliferation marker Ki67 (red) and the nuclear stain Hoechst 33342 (blue). **(D)** Confocal two-dimentional gallery of single niche stained for Pdpn (green) and Ki67 (red). 40 series stacks were collected from the bottom to the top with an optical slice thickness of 1 μm. The last 5 layers of images have been removed to conserve space. **(E)** Representative image of the niche acquired from Z stack. DF: differentiation. **(F)** Confocal three-dimentional semispherical niche structure (stack of 40 optical sections) stained for Pdpn (green) and Hoechst 33342 (blue). Confocal microscope images were obtained with voxel size of 210(x)-210(y)-40(z) μm. Scale bar: 10 μm (A, B), 20 μm (C, D).

Niche structure formation has been first observed in holoclones derived from cell lines of normal tissues and resembles “rim-like niche” [3, 4]. In our study, we would like to stress that all tested NSCLC cell lines contained holoclone cells, some of which were able to form niche structures (data not shown). Single LuCSC-holoclones, after 96 hrs cultivation, displayed homogeneous cell morphology. However, in the epithelial cobblestone cells, towards the center of the colony, approximately 50 cells displayed well-defined spindle-like morphology, suggesting the putative initiation of niche formation (Figure 2A). Within 3 months of growth and differentiation, multiple niches gradually formed and spread throughout the colony from the middle towards the edge (Figure 2B).

In single niches the AT1 marker Podoplanin (Pdpn), a transmembrane mucin-like glycoprotein, was highly expressed in two different cell populations: niche border cells and a chain of cells inside the niche. Pdpn^+^ cells frequently overlapped with the cells expressing Ki67, a marker of mitotic cells; however, cells with cuboidal morphology had weak Pdpn expression, and were often Ki67 negative or quiescent (Figure 2C). Confocal analysis was further applied to gain insight of LuCSC niche architecture. The spatial organization of the LuCSC niche resembled a cylinder with similar sizes on the basal and apical surface layers. For the first time we observed that LuCSC-holoclones initiated the formation of rim-niches from a basal lamina cell population (Figure 2D, top panel). Inside of the niche LuCSCs created pseudo-alveoli structures, which comprised AT1 spindle (Pdpn^high^) and AT2 cuboidal (Pdpn^low^) with lipid droplets cells (Figure 2D, middle panel and 2E). We analyzed other niches under the same circumstances which demonstrated similar architecture, with variations of pseudo-alveoli structure form and depth (data not shown). Three-dimensional reconstruction of single niche demonstrates that hemi-spherically organized cells are directed down the middle (Figure 2F).

At low cell density, consistent with mRNA expression, immunofluorescence staining indicated double lineage marker expressions in 2^nd^ DF cells (Supplementary Figure 2A). Pdpn was strongly expressed in arch-like cells, while cuboidal cells demonstrated AT2-specific SPC1 droplet staining. Surprisingly, progeny cells, at high cell density, lost Pdpn expression and all were converted to AT2-like cuboidal pneumocytes (Supplementary Figure 2B). AT2/AT1 double positive cells have been recently observed *in vivo*, where this phenotype was suggested to be associated with the ‘priming’ or increased plasticity of AT2 progenitor cells [24].

### LuCSCs and progeny cells in monolayer and alveospheres inversely express EGFR/MIG6 genes

**Figure 3:**
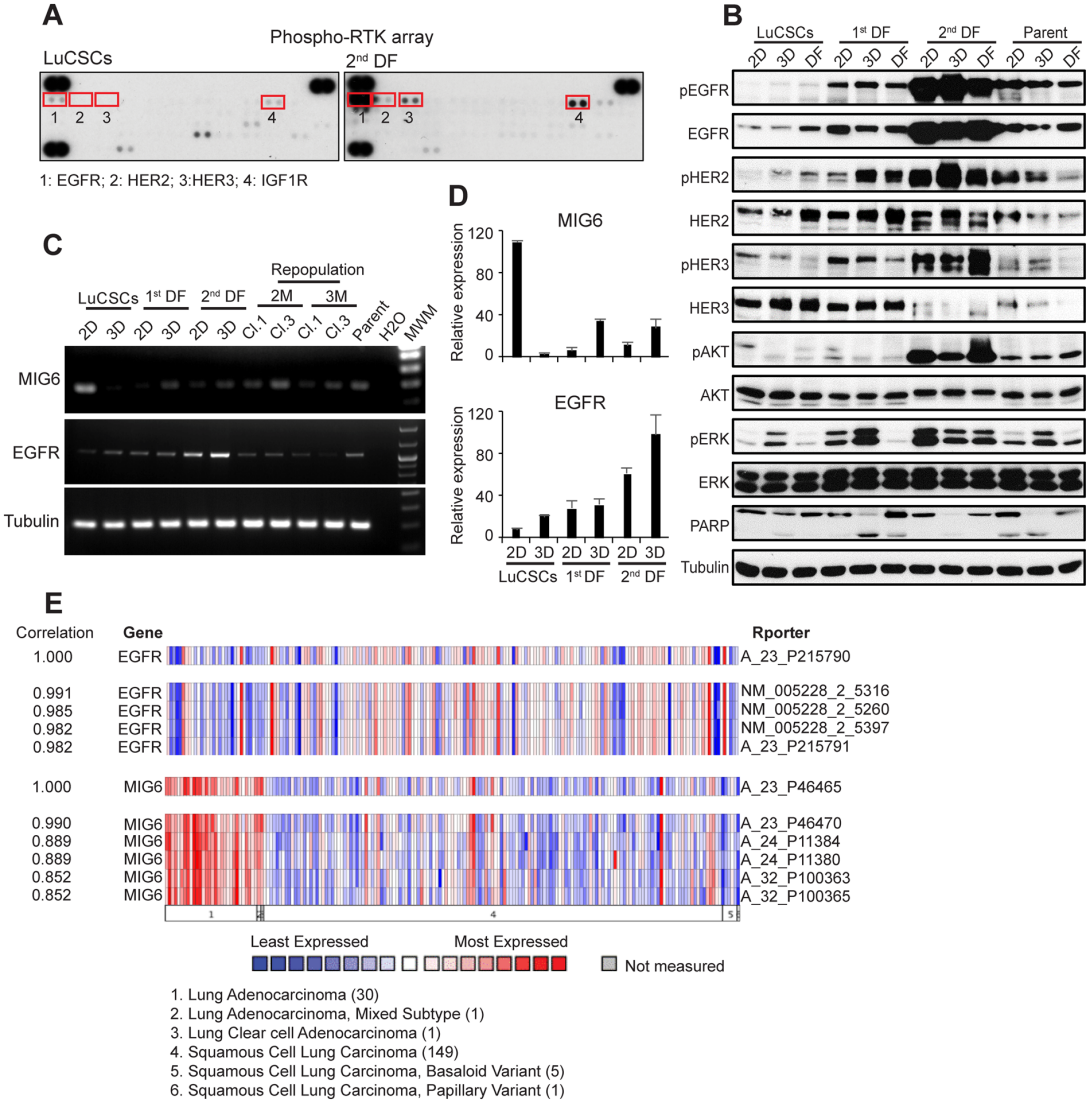
EGFR and MIG6 are inversely expressed in LuCSCs and their progeny **(A)** Phospho-receptor tyrosine kinase array was used to simultaneously detect the relative phosphorylation of 49 different RTKs on LuCSCs and 2^nd^ DF cells. **(B)** Monolayer cells (2D), alveospheres (3D) and cells after alveosphere differentiation (DF) from three distinct subclones as well as parent cells were lysed and prepared for western blot analysis of EGFR and downstream signaling proteins involved in proliferation and apoptosis. Tubulin served as a loading control. **(C)** Gene expression of EGFR and MIG6 in 2D and 3D LuCSCs, 1^st^ DF and 2^nd^ DF cells as well as the reprogrammed clones. Conventional PCR was used for evaluation. **(D)** Histogram quantification dialog of EGFR and MIG6 mRNA expression values. **(E)** Heatmap of inverse expression profiling of EGFR and MIG6, observed in 30 samples of the lung adenocarcinomas in comparison with 149 squamous cell lung carcinomas (https://www.oncomine.org TCGA lung).

To determine the receptors involved in self-renewal and differentiation potential of colony forming cells, we used phospho-receptor tyrosine kinase array and identified the upregulation of EGFR, HER2, HER3 and IGF-R1 in 2^nd^ DF cells relative to LuCSCs (Figure 3A). Later we analyzed EGFR-module signaling in parent cells and individual cell clones under 2D, 3D and differentiation conditions (Figure 3B). The phosphorylated proteins of the EGFR signaling pathway, including EGFR, dimerization partner HER2, as well as downstream signaling molecules AKT and ERK1/2, were barely detected in LuCSCs, but gradually increased and displayed their highest expression in 2^nd^ DF cells. All these signaling proteins showed moderate expression in heterogeneous parent cells. 2^nd^ DF cells demonstrated unique apoptosis resistance in cell culture, due to EGFR overactivation and strongest expression of AKT, even under serum-depleted conditions (data not shown). However, 3D-alveosphere conditions facilitated PARP cleavage among all cell lines, suggesting the induction of apoptosis upon overgrowth.

Our previous publications indicated that MIG6 can negatively modulate EGFR signaling [14, 16]. Apparently, MIG6 gene expression was high in LuCSCs, but barely detectable in cell clones with high EGFR status (Figure 3C and 3D). The inverse expression of MIG6 and EGFR in LuCSCs and the progeny revealed a new mechanism controlling self-renewal program and differentiation. We have conducted a study where ONCOMINE microarray data was analyzed to evaluate EGFR gene expression in NSCLC tumors [25]. Low EGFR expression was observed in lung adenocarcinomas compared to squamous cell lung carcinoma, especially if simultaneously MIG6 expression was high (Figure 3E).

### EGFR controls invasive behavior of the poorly differentiated progeny derived from LuCSCs

**Figure 4:**
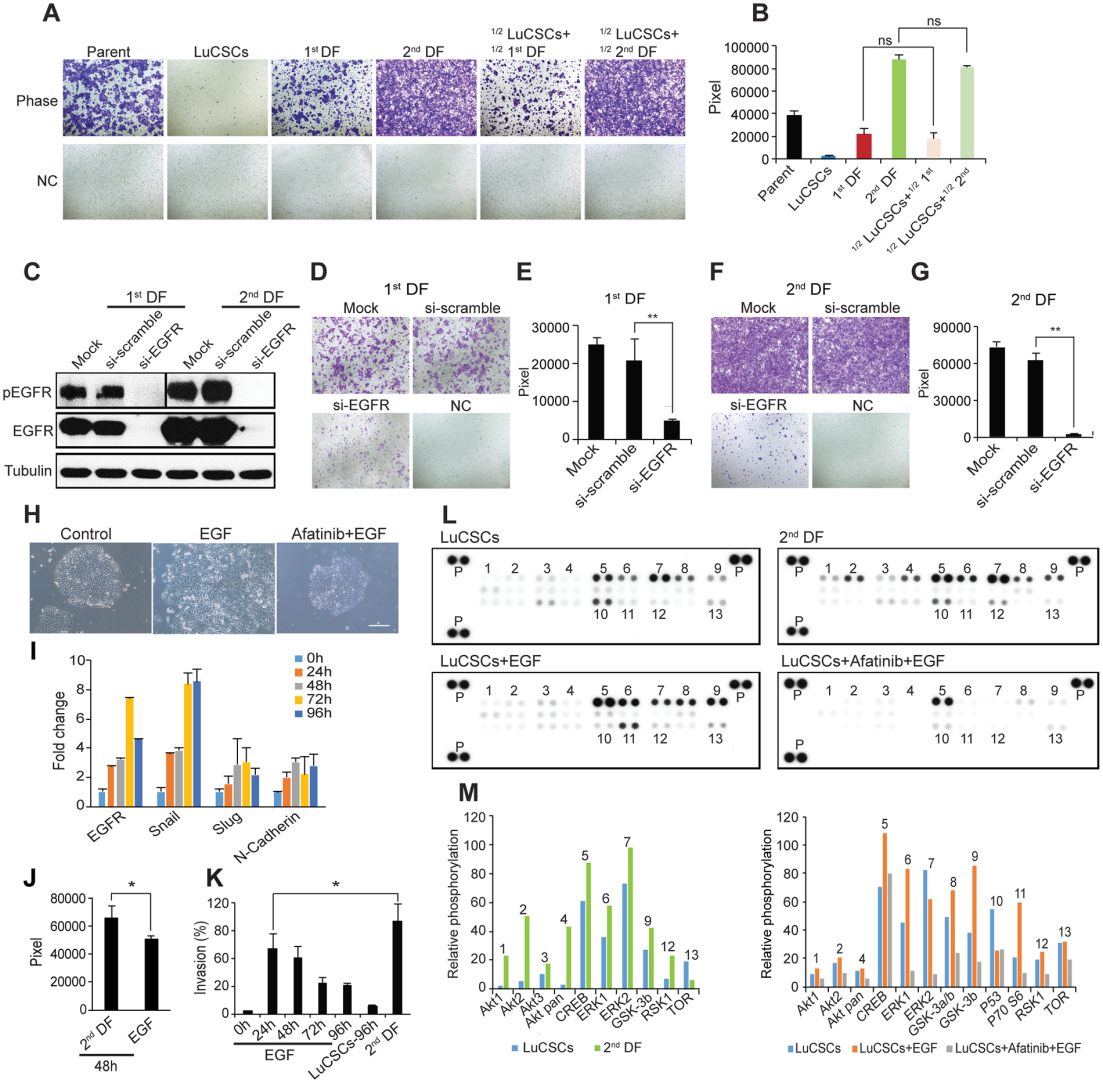
EGFR ligands induce LuCSC EMT-like differentiation **(A)** Boyden chamber evaluation of NCI-H1568 parent cells and subclones, as well as mixed populations of LuCSCs with progeny cells. ½ LuCSCs + ½ 1^st^ DF: LuCSCs and 1^st^ DF cells were mixed at equal proportions and then added into the Boyden chamber inserts. ½ LuCSCs + ½ 2^nd^ DF: LuCSCs and 2^nd^ DF cells were mixed at equal proportions and then added into the Boyden chamber inserts. NC, negative control. **(B)** Quantification of cell invasiveness shown in (A). ns, not significant. **(C)** 1^st^ and 2^nd^ DF progeny cells 72 hrs after transfection with siRNA-EGFR and siRNA-scramble were lysed and protein was prepared for western blot analysis of pEGFR and EGFR. Tubulin served as a loading control. Representative images show reduced cell invasiveness of 1^st^ DF **(D)** and 2^nd^ DF (F) cells due to EGFR knockdown compared to control cells. NC, negative control. **(E** and **G)** Quantification of cell invasiveness shown in (E) and (G). ^**^P < 0.01. **(H)** Representative images of LuCSCs stimulated with EGF (5 ng/ml) with/without 3.3 μM Afatinib pretreatment. Scale bar: 20 μm. **(I)** Real-time PCR analysis of EGFR and mesenchymal markers. Gene expression levels of LuCSCs were measured upon EGF stimulation at different time points as indicated. **(J)** Comparison of invasive activity of LuCSCs after stimulation with EGF and conditioned medium derived from 2^nd^ DF cells (20% as chemoattractant) for 48 hrs. ^*^P < 0.05.**(K)** Quantification of invasive activity of LuCSCs upon EGF stimulation at different time points as indicated. The values were indicated in % of control 2^nd^ DF cells. ^*^P < 0.05. **(L)** Whole-cell lysates from LuCSCs, 2^nd^ DF cells, EGF stimulated LuCSCs with/without Afatinib (3.3 μM) pretreatment were collected for human phospho-kinase antibody array analysis. Each membrane contains kinase specific (number indicated) and positive control (P). **(M)** Relative phosphorylation of spots was quantified by normalizing pixel density of the positive control to 100. Each bar is represented as mean of duplicate spots.

Invasion and metastasis are the most insidious and life-threatening aspects of cancer [26]. NCI-H1568 parent cells were initially checked to possess high capability of invasion (Figure 4A, top left). After sub cloning we wondered how invasiveness was associated with defined clones. Strikingly, we found that epithelial LuCSCs had no invasion capacity, whereas 2^nd^ DF cells exhibited a greater than 2 and 4 fold increase in invasion compared to parent cells and 1^st^ DF cells, respectively (Figure 4A, middle panel and Figure 4B). Our data argues against the EMT nature and aggressive behavior of CSCs [27, 28]. To address the potential influence of LuCSCs on their progeny invasiveness, we co-cultured LuCSCs with 1^st^ and 2^nd^ DF cells at equal proportions for 36 hrs. Surprisingly, the invasion was increased approximately 2 fold because of co-culture of either 1^st^ or 2^nd^ DF cells with LuCSCs (Figure 4A, two right panels).

Next, we examined whether endogenous EGFR signaling plays a role in mediating the invasive behavior of poorly differentiated adenocarcinoma progeny. To this end we utilized EGFR-siRNA and observed effective EGFR knockdown in the highly invasive 1^st^ and 2^nd^ DF cells (Figure 4C). Notably an equal inhibition of pEGFR was seen in both cell lines (Figure 4C). As expected, the invasiveness of both cell lines was almost abrogated by EGFR-siRNA specific inhibition (Figure 4D-4G). Taken together, our data demonstrated EGFR signaling is critical for the invasiveness of 1^st^ and 2^nd^ DF cells and possibly for the initiation of paracrine mediated LuCSC differentiation into aggressive mesenchymal cells.

We further identified EGFR ligands in the role of stimulating LuCSC differentiation. As expected, EGF overnight application induced profound LuCSC differentiation (Figure 4H, middle); however, pretreatment of LuCSCs with the EGFR inhibitor Afatinib abrogated this stimulatory effect (Figure 4H, right). The blocking effect by Afatinib confirmed that EGFR activation is critical for the initiation of LuCSC differentiation. Real-time PCR was employed simultaneously to examine the expression of a selection of genes, with potential relation to mesenchymal cells. The upregulation of EGFR, snail, and slug, together with N-cadherin, went along with the stimulation time (Figure 4I). We also observed that other EGF family ligands (Amphiregulin, TGFα, HB-EGF and Heregulin-β1) and cytokines (IL6 and Oncostatin M), as well as the serum free medium optimized for the culture of mammospheres, all triggered LuCSC differentiation towards a mesenchymal phenotype (Supplementary Figure 3A). Moreover, short time incubation of the ligands EGF, TGFα and HB-EGF with their specific antibodies completely abrogates the stimulatory effect (data not shown). EGF which induced phenotypical changes did also enhance LuCSC invasiveness, but to a significantly lesser extent than conditioned medium derived from 2^nd^ DF cells (Figure 4J). Additionally, the invasive ability of LuCSCs after EGF stimulation was inversely correlated to the cultivation time (Figure 4K).

Later we used human phospho-kinase arrays to analyze and understand how cells recognize and respond to changes in their environment. The data revealed a range of signaling molecules that were phosphorylated in response to EGF treatment in comparison with Afatinib blockade, and significant ones were highlighted by numbers (Figure 4L). In LuCSC-holoclones, AKT1/2/3 was weakly expressed and no activation (AKT pS473) was observed after EGF stimuli, indicating at EGFR silent and stemness states EGFR-PI3K-AKT pathway does not play a significant role for survival and metabolic homeostasis. However, the AKT pathway was strongly activated in progeny 2^nd^ DF cells (Figure 4M). Upon EGF stimulation, pCREB is increased in the LuCSCs in conjunction with β-catenin, Stat3 (Supplementary Figure 3B-3D), GSK3 (a/b) and p70S6 activations. However, pretreatment with Afatinib dramatically inhibited these protein activities, except CREB which was partially inhibited to basal level. Conversely, P53 was downregulated after EGF stimulation (Figure 4M). Overall, data indicates that growth response of LuCSCs mediated by EGF stimulation partially mimics early stage of differentiation.

### Sensitivity of LuCSCs and their aggressive progeny towards chemo drugs and EGFR-TKIs

**Figure 5:**
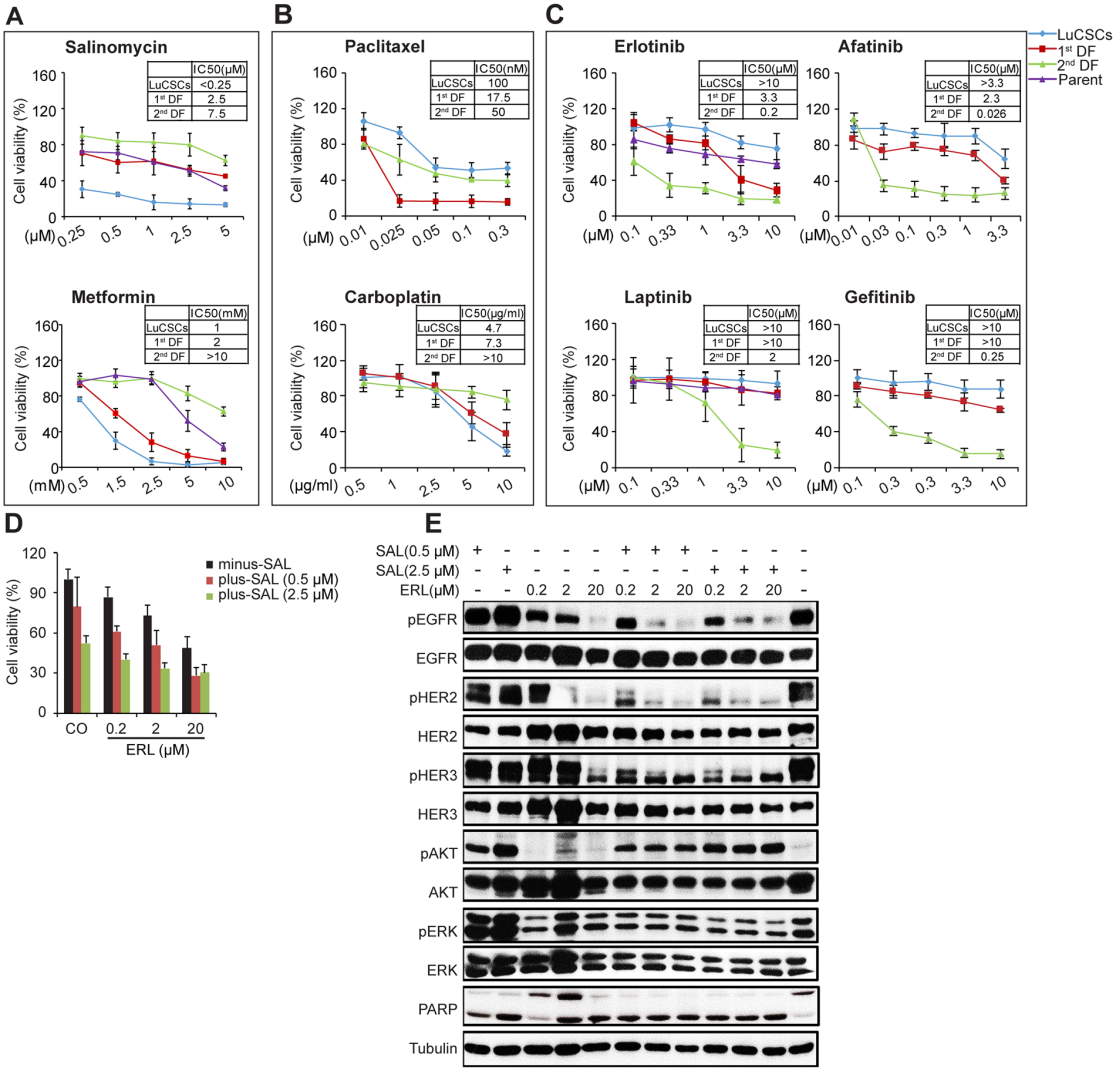
Sensitivity of LuCSCs and their progeny cells to cytotoxic drugs, EGFR-TKIs, Metformin and Salinomycin **(A-C)** Response of NCI-H1568 parent cells (purple), LuCSCs (blue), 1^st^ DF cells (red) and 2^nd^ DF cells (green) to different drug treatment as indicated. The viable cells were assessed by CellTiter-Glo cell viability assay. The data of the survival curves were plotted as percentages of DMSO controls. Each experiment consists of 4 replicates for each point and the plotted data represent the average mean ±SD of three independent experiments. **(D)** Cell viability of NCI-H1568 parent cells after combined treatment with various concentrations of Salinomycin and Erlotinib as indicated. The viable cells were assessed by CellTiter-Glo cell viability assay. The data of the survival curves were plotted as percentages of DMSO controls.**(E)** EGFR signaling evaluation of NCI-H1568 parent cells after combined treatment with various concentrations of Salinomycin and Erlotinib as indicated.

Putative CSCs have been shown to be associated with refractoriness to chemotherapy, to some extent explaining why tumors initially shrink after conventional treatment, then later reoccur [29]. Hence, drug sensitivity of cells isolated from these three types of colonies was evaluated by the application of chemotherapeutic drugs (Paclitaxel and Carboplatin), TKIs (Afatinib, Lapatinib, Erlotinib and Gefitinib) and putative CSC killers (Salinomycin and Metformin). Among the entire concentration range tested, the survival rates of LuCSCs were significantly higher than those of 1^st^ and 2^nd^ DF cells when treated with Paclitaxel and TKIs (Figure 5B top and 5C). However, LuCSCs were highly sensitive to Salinomycin and Metformin, as indicated by the low concentrations of 100 nM and 1 mM, causing 50% cell growth inhibition, respectively (Figure 5A). Single agent treatment with TKIs induced a substantial cell growth inhibition on 2^nd^ DF cells (Figure 5C). Among all the tested drugs, 1^st^ DF cells showed their highest sensitivity to Paclitaxel, with IC_50_ at 50 nM. 2^nd^ DF cells highly express EGFR to constitutively activate signaling via autocrine stimuli, contributing to the drug sensitivity of aggressive progeny cells to EGFR-TKIs.

We hypothesized that, in parent cells, TKIs target rapidly proliferating cells (e.g. 1^st^ and 2^nd^ DF cells) but spares slow-dividing LuCSCs which represent the chemo- and TKI resistance. Cell viability assay indicated that parent cells were very resistant to Erlotinib and Lapatinib (IC_50_ > 20 μM), and resistant to Salinomycin and Metformin (Figure 5A and 5C). Combinatorial treatment of parent cells with various concentrations of Salinomycin and Erlotinib enhanced growth inhibition over that observed with either single agent (Figure 5D). The beneficial effect of combined treatment is evident for all combinations with a more significant effect observed when a higher dose (2.5 μM) of Salinomycin was used.

To further estimate EGFR signaling which might correlate with the observed growth inhibition, we examined the effect of Erlotinib and Salinomycin alone or in combination on the activation state of several key regulators involved in the EGFR signaling pathway (Figure 5E). 48 hrs treatment with increasing concentrations of Erlotinib resulted in a gradual reduction in the activated forms of EGFR, HER2 and HER3, which were further reduced in the face of co-treatment with Salinomycin. Suppression of pERK1/2 was only observed with dual treatment, and with weak inhibitory effect. At this check point AKT, an anti-apoptotic protein, was strongly activated in a dose dependent manner upon Salinomycin monotreatment. Co-exposure to Erlotinib, which alone didn't induce AKT activation, decreased Salinomycin-triggered AKT phosphorylation. Additionally, single-agent Salinomycin or Erlotinib both elicited a dose-dependent activation of PARP, whereas the pronounced apoptosis activation evident under combinatorial treatment was no longer affected by dose.

## DISCUSSION

Highly tumorigenic and self-renewing cells, with properties like CSCs, have been reported in both SCLC and NSCLC using CD133^+^, CD44^+^, CD24^+^, and CD133^+^ cell surface markers or high ALDHA1 activity [21, 30–33]. However, neither the characteristics of CSC morphology or lineage-specific differentiation nor their interactions with the niche are known. In our study, we used NSCLC cell lines as the *in vitro* tumor models to translate cellular heterogeneity into tractable populations to understand the cellular origins of lung cancers and drug resistance.

Three-major hierarchical organized cell populations, named as LuCSCs, 1^st^ and 2^nd^ DF cells, were isolated in relation to stem and lineage-specific marker expressions. We identified that LuCSC-holoclones were lineage-naïve and with the ability to grow indefinitely in culture. They could undergo spontaneous and inducible differentiation in 2D-monolayer to create aggressive progeny expressing AT2/AT1/Club markers, suggesting their origination from putative bronchioalveolar bipotent stem/progenitor cells. Gene expression profiling demonstrated that LuCSCs were EpCAM^+^/CD44^+^/BIMI1^+^/Nanog^+^/β-catenin^+^/IL6^+^, while these genes are specificaly transcribed in stem cells and iPSCs as shown in many publications. We extrapolate that transformation turned stem cells into LuCSCs. The bipotent LuCSCs hijack stemness, sustain malignancy and preserve the capability to be differentiated into aggressive descendants. Alveosphere culture also revealed to be a good approach to initiate LuCSC differentiation into lineage specific progeny. Under this condition, AT2 cells were able to trans-differentiate into Club cells with CC10 expression.

Although the existence of the CSC niche is accepted, precise knowledge of its 3D architecture remains unknown. These rim-cell niches identified in our lung cancer cell model highly resemble the niches observed in normal tracheal epithelial basal cells and in holoclones of the hair follicles [4, 5]. In human lungs, fibroblasts were shown to maintain AT2 stem cell property by providing single cell fibroblast niches [34]. Further evidence also suggests that *in vivo* there are at least two populations of stromal cells in the alveolar niche, and only one of which, mesenchymal, promotes alveolar organoid growth [35, 36]. One new observation reported here is that LuCSC-holoclones initiate the formation of rim-niches from a basal lamina cell population, which potentially functions as feeder cells. These mesenchymal cells could further produce paracrine signals to transiently expand the progenitor pool where LuCSCs were indefinitely preserved, or in other words, protected from differentiation in cell culture condition. Inside of the niches pseudo-alveoli structures were generated, where presumably mesenchymal cells and extracellular matrix orchestrated malignant AT2/AT1 lineage formation. Future studies will need to test the functional significance of the association between LuCSCs and mesenchymal cells in holoclone niches.

Numerous publications indicate that EMT is a key program to generate CSCs. Our data sheds light on a new understanding of LuCSCs. The LuCSC-holoclones were EpCAM^+^ (morphologically epithelial), and negative for classical EMT genes AXL, CD10, Zeb1 and MMP1 that are involved in motility and invasive behavior of mesenchymal cancer cells [37, 38]. LuCSC-holoclones weakly expressed Twist2, however, the RNA-transcription was dramatically activated in their alveospheres. We extrapolate that Twist2 expressing LuCSCs were cells committed for EMT at the edge of colonies that accompany morphology changes. Nevertheless, they do not demonstrate any invasive activity. It is challenging to preserve LuCSCs from epithelial transition in culture or cell sorting. In this respect, we speculate that the sorted tumor initiating cells used in many publications have already been differentiated into aggressive descendants, most likely 1^st^ DF cells, to be tumorigenic or invasive. Mechanistically, the regulation of LuCSC transition from self-renewal to differentiation could be highly connected to the activation of EGFR signaling and the inhibition of MIG6. These inverse regulations are well demonstrated in clinical lung cancer samples [39]. For the first time we observed that tumor suppressor MIG6 is highly expressed in LuCSCs and downregulated in the aggressive progeny. There has been some data indicating that MIG6 expression is regulated epigenetically through promoter methylation or histone deacetylation inhibition [40, 41]. Additionally, we cannot exclude the possibility that promoter hypermethylation of EGFR silences its expression in LuCSCs, and demethylation in progeny cells drives its expression [42].

The EMT has been implicated in resistance development, which is not the case in our cell line model. 1^st^ DF and 2^nd^ DF cells displayed typical EMT traits, however, 2^nd^ DF cells were extremely sensitive to EGFR-TKIs and 1^st^ DF cells to Paclitaxel as well. Both act as inhibitors of highly proliferative cell populations. Aggressive 2^nd^ DF cells (almost no stemness) possess exclusive growth efficiency in serum-depleted culture condition due to high expressions of EGFR and its ligands as well as AXL/GAS6, providing them autologous signal for proliferation and survival. Surprisingly, IC_50_s of LuCSCs to Erlotinib and Afatinib were more than 20 μM, which exceeded their solubility in culture medium. We speculate that EGFR silence in LuCSCs contributes to the failure of NSCLC mono-therapy, even though adenocarcinomas are driven by overexpressed, mutant or wild type EGFR. Additionally, LuCSCs and 1^st^ DF cells (retaining stemness partially) were exclusively sensitive to antibiotic Salinomycin and AMPK agonist Metformin. Indeed, several approved antibiotics have already shown effects on putative CSC survival in preclinical models and clinical studies via reduction of stemness properties [43]. Mitochondrial health is fundamental for the maintenance of CSCs and can be targeted for cancer therapy. The ability of Metformin to induce autophagy, AMPK activation, and cell death can be attributed to decreased energy production, due to a partial, but coordinated compromise of glycolysis and mitochondrial function of the respiratory chain [44–47]. To expose the clinical relevance of our study, we further compared the IC_50_s of our tested drugs and their maximum plasma concentrations (C_max_) in patients [48]. Salinomycin is currently an experimental medication, but has not gone to clinical trials. Overall, (1) IC_50_s of 1^st^ DF and LuCSCs to TKIs are higher than C_max_, which perfectly explains why TKIs can only cause tumor shrinkage, but not elimination. (2) Although we didn't evaluate such a high dose of Carboplatin (C_max_=50 μg/ml), 2^nd^ DF cells are clearly not responsive to all the tested concentrations (high as 10 μg/ml). (3) The plasma C_max_ of Paclitaxel appears to be much higher than IC_50_s of all clones. Our previous work demonstrated that other NSCLC cell lines (e.g. NCI-H1975 and HCC95) were less responsive to Paclitaxel [45], so establishing a link between the molecular subtype and drug response will enable better stratification of patients for improved therapeutic strategies. (4) Due to the short-term exposure and supraphysiologic glucose in cell culture, mM concentrations of Metformin are often required to induce anti-cancer affects in *in vitro* studies, whereas in humans the therapeutic dose is μM. Wahdan-Alaswad et al. have shown that Metformin treatment of cells for weeks or months, as is required for clonogenicity assays, anti-cancer activity can be detected using doses in the μM range [49].

Thus, successful lung cancer treatment requires combinatorial application or a novel approach to target both LuCSCs and differentiated tumor cells. We have previously shown that combination of Salinomycin and Metformin was best for treatment of alveospheres consisting of LuCSCs and other differentiated cell populations [45]. In the current study we tested our hypothesis by treating the parental heterogeneous NCI-H1568 cells with Salinomycin and Erlotinib alone or in combination. The results clearly demonstrated that either Salinomycin or Erlotinib eliminates different cell populations, via alternative pathway of inhibitions. Salinomycin led to cell death without inhibiting EGFR signaling and surprisingly, pAKT and pERK1/2 were increased. However, Erlotinib treatment lead to classical programmed cell death via EGFR/ERK1/2 signaling axis inhibition and Caspase3-PARP activation in progeny cells [44]. Consistent with the elevated stem cell marker expression upon Erlotinib treatment, combinatorial treatment eradicated the parental cell population at much lower concentrations of both drugs. As suggested, this combinatorial setting has already been conducted in NSCLC patients in several clinical trials. For example, the phase 3 FASTACT-2 applied Erlotinib intercalated with chemotherapy (Gemcitabine plus Carboplatin/Cisplatin) into patients with advanced NSCLCs. Data had shown that the intercalation regimen improved progression free survival in Asian patients bearing the EGFR mutated form, but these results were not yet translated into clinical practice [50]. We would like to stress that EGFR naivety in LuCSCs is important for understanding their resistance to TKIs. Targeting CSCs with mitochondrial respiratory chain modulators/inhibitors, AMPK agonist (Metformin/Phenformin) or antibiotics (Erythromycin or Salinomycin) would be an option to overcome treatment resistance associated with EGFR naïve LuCSC; however, it still presents a big challenge due to lack of knowledge about mechanisms of their actions. Establishing LuCSC-holoclone cell lines, from fresh biopsies of pre/after EGFR-TKI treated NSCLC patients, will allow better understanding of the cellular origin of lung tumor heterogeneity, and all these will lead to improved therapeutic strategies. Our results open a new chapter in the development of novel approaches to overcome TKI resistance phenomena in lung adenocarcinoma treatment.

## MATERIALS AND METHODS

### Cell lines and chemicals

The human NSCLC cell lines, NCI-H1568 and A549 were obtained from the American Type Culture Collection. HCC95, NCI-H3122, NCI-H1650, HCC827, NCI-H1975, normal airway epithelial cell line (SALE) and KRAS^G12D^ transformed SALE cells (SALER) were kindly provided by Dr. Thomas Roman. Cancer cells were cultured in RPMI-1640 medium supplemented with 10% FBS, L-glutamine, 100 U/ml penicillin and 50 μg/ml streptomycin. Cell lines were identity-verified by STR analysis and certified as mycoplasma-free within 6 months of the experiments reported. All cell lines were authenticated using the Stem-Elite ID System (Promega, Madison, WI, USA).

Tyrosine kinase inhibitors Gefitinib, Lapatinib, and Erlotinib were purchased from Vichem Chemie. Chemotherapeutic agents Paclitaxel, and putative stem cell killers Metformin and Salinomycin were purchased from Sigma. Afatinib was purchased from SelleckChem, Carboplatin was purchased from Santa Cruz.

### siRNA-cell line transfections

Cells were plated at 60% to 80% confluence in 6-well plate with antibiotic-free culture medium. Transfection of 21-nucleotide siRNA specific for EGFR (Thermo Fisher, #1299001) as well as a corresponding scrambled siRNA (Thermo Fisher, #12935-200) was carried out using Lipofectamine™ RNAiMAX (Invitrogen) according to the manufacturer's protocol.

### Proliferation assays

LuCSCs, 1^st^ DF and 2^nd^ DF cells were seeded at 1000, 2000 and 5000 cells/well. Proliferation was measured 2, 4, 6, and 8 days after cell seeding using CellTiter-Glo luminescent cell viability assay (Promega), following manufacturer's instructions. Experiments were set up in four replicate wells and repeated thrice. Opaque-walled 96-well plates with clear bottoms were used for cells culture.

### Western blot

Protein samples were electrophoresed using 7.5 or 10% SDS–PAGE and performed as previously described [45]. Membranes were incubated with the primary antibodies against pEGFR Y1173 (Cell Signaling, #4407), EGFR (Transduction Laboratories, E12020), pHER2 Y1248 (Cell signaling, #2247), HER2 (Millipore, #06-562), pHER3 Y1289 (Cell signaling, #4791), HER3 (Millipore, #05-390), pERK1/2 (Cell Signaling, #9101), ERK1 K23 (Santa Cruz, sc-94), pAKT (Cell Signaling, #9271), AKT1/2/3 H-136 (Santa Cruz, sc-8312), PARP (Cell Signaling, #9542), Tubulin (Sigma, T9026), Secondary HRP-conjugated anti-rabbit (Bio-Rad) and anti-mouse (Sigma) antibodies were used and detection was done using an ECL reagent.

### Alveospheres formation assay

Monolayer cells from individual clones were seeded in ultra-low adherent 6-well plate and grown in serum-free MammoCult™ Medium: DMEM/F12 (1:1, Gibco) supplemented with 30% Glucose (Sigma, G8270), Hepes (Serva, 25245), Progesterone (Sigma, P8783), Putrescine (Sigma, P5780), B27 (Gibco, 17504), EGF (Peprotech, AF-100-15), FGF2 (Sigma, F0291), ITSS (Roche, 110745470), Heparin (Sigma, H3149) and NaHCO3 (Invitrogen, 25080-060).

### Boyden chamber invasion assay

50,000 cells resuspended in 350 μl of serum-free medium were seeded into the Matrigel-coated Boyden chamber inserts (BD Biosciences). In the bottom chamber 750 μl medium with or without 10% FCS served as chemoattractant or negative control, respectively. Cells were permitted to migrate for 36 hrs, then fixed and stained with crystal violet. Pictures were taken on a Zeiss Observer. A1 microscope. The value of the migrated cells was calculated from at least three wells for each experiment group and analyzed with the Photoshop CS3 extended measurement feature.

### Immunofluorescence assay

Cells were seeded onto 12 mm coverslips in 24-well dishes and allowed for attachment. Cells were fixed in methanol, blocked in 3% BSA, and incubated with the following primary antibodies: Podoplanin (Santa Cruz, sc-376695), SPC1 (Santa Cruz, sc-13979), Ki67 (Abcam, ab15580), Nanog (Abcam, ab21624), β-catenin (Transduction Laboratories, #610154). After incubation with secondary antibody Alexa Fluor 488-conjugated goat anti-mouse (Jackson Immunoresearch, #115-545-003) or Alexa Fluor 594-conjugated goat anti-rabbit (Jackson Immunoresearch, #111-585-144), nuclei were stained with Hoechst 33342. Coverslips were mounted in Prolong Gold Antifade Reagent (Cell Signaling, #9071). Image acquisition was carried out using a Zeiss Axioplan2 microscope and the MediaView software.

### Flow cytometry

1-2 × 10^6^ cells grown in 6-well plates were trpsinized and fixed with 70% ice cold ethanol followed by 30 minutes incubation at 4 °C. Cells were stained with 0.1% Triton X-100, 10 g/mL PI (Molecular Probes), and 100 μg/mL DNase-free RNase A in PBS at room temperature in the dark for 30 minutes. Cell cycle distribution was determined by FACScan flow cytometer (BD Biosciences) and analyzed by Cell Quest software (BD Biosciences).

### Phospho-kinase array

The human Phospho-receptor tyrosine kinases array (R&D Systems, ARY001B), Proteome Profiler Human Phospho-Kinase Array Kit (R&D Systems, ARY003B) and Proteome Profiler Human Phospho-MAPK Array Kit (R&D Systems, ARY002B) were used following manufacturer's instructions. Spot densities were analyzed using the AIDA software. The average density of duplicated spots was determined and normalized to the controls.

### Confocal microscopy

Slides were viewed using Zeiss LSM 780 confocal microscope equipped with 405-, 488-, and 543-nm laser lines and a GaAsp detector. Images were collected using 40× objectives acquired through Z-stack acquisition, with an increasement of 1 μm between image frames and processed using the Zeiss software. The AxioCamMRm camera was utilized to capture images. Cells expressing both Ki67-Red and Podoplanin-Green were selected, and Z-stack acquisition was performed. The LuCSC niche whole mounts were scanned from the basal layer 0 to apical surface layer 40. The 3D organization and size of the rimmed-niche were displayed in X-210 x Y-210 x Z-40 μm. Images were displayed as maximum intensity projections and subsequently analyzed for co-localization using 2D/3D cyto-fluorograms and fluorescence intensity line profiles obtained with the use of the Zen 2016 imaging software.

### Gene expression analysis

RNA was extracted using the RNAeasy kit (QIAGEN) and 5 μg isolated total RNA was reverse-transcribed into cDNA as template for PCR amplifications. Semi-quantitative PCR was performed using the ReadyMix™redtaq™ PCR reaction mix (Sigma), and PCR products were subjected to electrophoresis in 2% agarose gels. Detailed primer sequences are in Supplementary Table 1.

### Real-time PCR

All quantitative PCR reactions (20 μl) were carried out in the StepOne™ plus Real-Time PCR system (Applied Biosystems) using Fast SYBR Green Master Mix (AB Applied Biosystems, Darmstadt, Germany). The 2 ^#x02C9;^ΔΔCT method was used to analyze the relative fold change in gene expression with hypoxanthine phosphoribosyl-transferase (HPRT) (Applied Biosystems) as an endogenous control. Data analysis and calculation of 95% confidence intervals was performed using the StepOne™ Software version 2.0. All specimens were evaluated in triplicates. Detailed primer sequences are in Supplementary Table 2.

### Statistical analysis

Data are represented as mean ±SD from three independent experiments unless stated otherwise. Statistical analysis was performed by one-way analysis of variance (ANOVA), and statistical significance was evaluated with the unpaired 2-tailed Student t test to assess difference between treated and control samples.

## SUPPLEMENTARY MATERIALS FIGURES AND TABLES



## Abbreviations

NSCLC: non-small cell lung cancer
CSCs: cancer stem cells
LuCSCs: lung cancer stem cells
EGFR-TKIs: EGFR tyrosine kinase inhibitors
EMT: epithelial mesenchymal transition
DF: differentiated
AQP5: aquaporin 5
SPA, SPC1: surfactant protein A
α1AT: alpha 1 antitrypsin
CC10: club cell secretory protein
Pdpn: Podoplanin
