# Correction: Lung cancer stem cells and their aggressive progeny, controlled by EGFR/MIG6 inverse expression, dictate a novel NSCLC treatment approach

**DOI:** 10.18632/oncotarget.28050

**Published:** 2021-08-17

**Authors:** Zhiguang Xiao, Bianca Sperl, Silvia Gärtner, Tatiana Nedelko, Elvira Stacher-Priehse, Axel Ullrich, Pjotr G. Knyazev

**Affiliations:** ^1^ Department of Molecular Biology, Max-Planck Institute of Biochemistry, Martinsried, Munich, 82152, Germany; ^2^ Department of Medicine, University of Rochester School of Medicine and Dentistry, Rochester, NY 14642, USA; ^3^ Department of Medicine III, Klinikum rechts der Isar, TUM, Munich, 81675, Germany; ^4^ Asclepius Institute of Pathology, Gauting, 82131, Germany; ^5^ Current address: DoNatur GmbH, Martinsried, Munich, 82152, Germany

**This article has been corrected:** In Figure 4A, the fourth panel and sixth panel of the ‘Phase’ row contain accidental image overlaps, as well as the first and second panels and the fourth and fifth panels of the ‘NC’ row. The corrected Figure 4, obtained using the original data, is shown below. The authors declare that these corrections do not change the results or conclusions of this paper.

Original article: Oncotarget. 2019; 10:2546–2560. 2546-2560. https://doi.org/10.18632/oncotarget.26817

**Figure 4:**
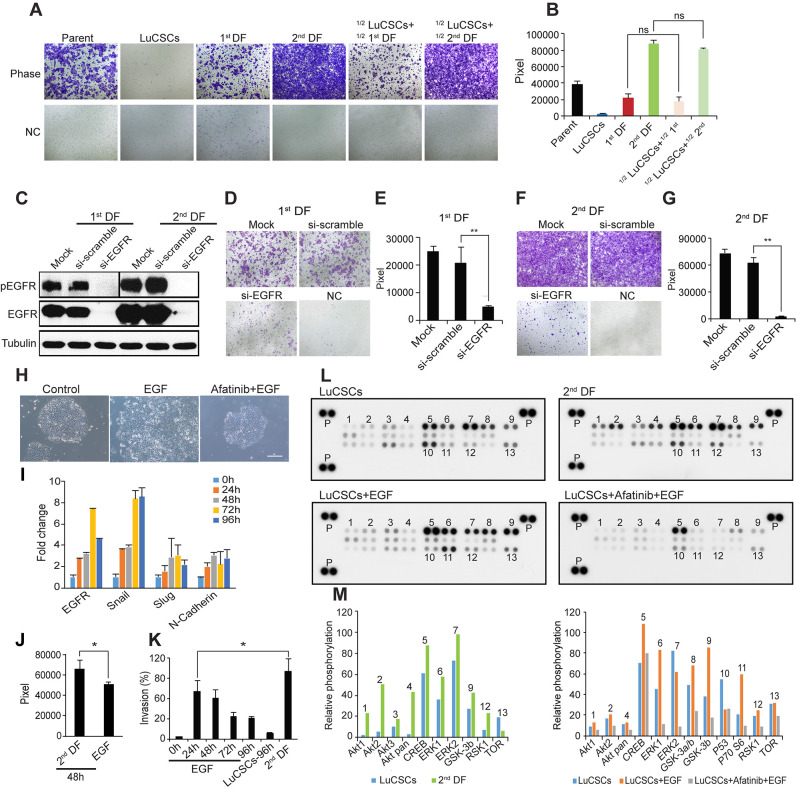
EGFR ligands induce LuCSC EMT-like differentiation. (**A**) Boyden chamber evaluation of NCI-H1568 parent cells and subclones, as well as mixed populations of LuCSCs with progeny cells. ½ LuCSCs + ½ 1st DF: LuCSCs and 1st DF cells were mixed at equal proportions and then added into the Boyden chamber inserts. ½ LuCSCs + ½ 2nd DF: LuCSCs and 2nd DF cells were mixed at equal proportions and then added into the Boyden chamber inserts. NC, negative control. (**B**) Quantification of cell invasiveness shown in (A). ns, not significant. (**C**) 1st and 2nd DF progeny cells 72 hrs after transfection with siRNA-EGFR and siRNA-scramble were lysed and protein was prepared for western blot analysis of pEGFR and EGFR. Tubulin served as a loading control. Representative images show reduced cell invasiveness of 1st DF (**D**) and 2nd DF (**F**) cells due to EGFR knockdown compared to control cells. NC, negative control. (**E** and **G**) Quantification of cell invasiveness shown in (E) and (G). **P < 0.01. (**H**) Representative images of LuCSCs stimulated with EGF (5 ng/ml) with/without 3.3 μM Afatinib pretreatment. Scale bar: 20 μm. (**I**) Real-time PCR analysis of EGFR and mesenchymal markers. Gene expression levels of LuCSCs were measured upon EGF stimulation at different time points as indicated. (**J**) Comparison of invasive activity of LuCSCs after stimulation with EGF and conditioned medium derived from 2nd DF cells (20% as chemoattractant) for 48 hrs. *P < 0.05. (**K**) Quantification of invasive activity of LuCSCs upon EGF stimulation at different time points as indicated. The values were indicated in % of control 2nd DF cells. *P < 0.05. (**L**) Whole-cell lysates from LuCSCs, 2nd DF cells, EGF stimulated LuCSCs with/without Afatinib (3.3 μM) pretreatment were collected for human phospho-kinase antibody array analysis. Each membrane contains kinase specific (number indicated) and positive control (P). (**M**) Relative phosphorylation of spots was quantified by normalizing pixel density of the positive control to 100. Each bar is represented as mean of duplicate spots.

