# Supplementary material for: Lung cancer stem cells and their aggressive progeny, controlled by EGFR/MIG6 inverse expression, dictate a novel NSCLC treatment approach
Source: Oncotarget. 2019 Apr 2;10(26):2546–60. doi: 10.18632/oncotarget.26817 (PMC6493460; doi:10.18632/oncotarget.26817)
Supplement: Supplementary file 1 [file oncotarget-10-2546-s001.pdf]

Lung cancer stem cells and their aggressive progeny, controlled by EGFR/MIG6 inverse expression, dictate a novel NSCLC treatment approach

SUPPLEMENTARY MATERIALS

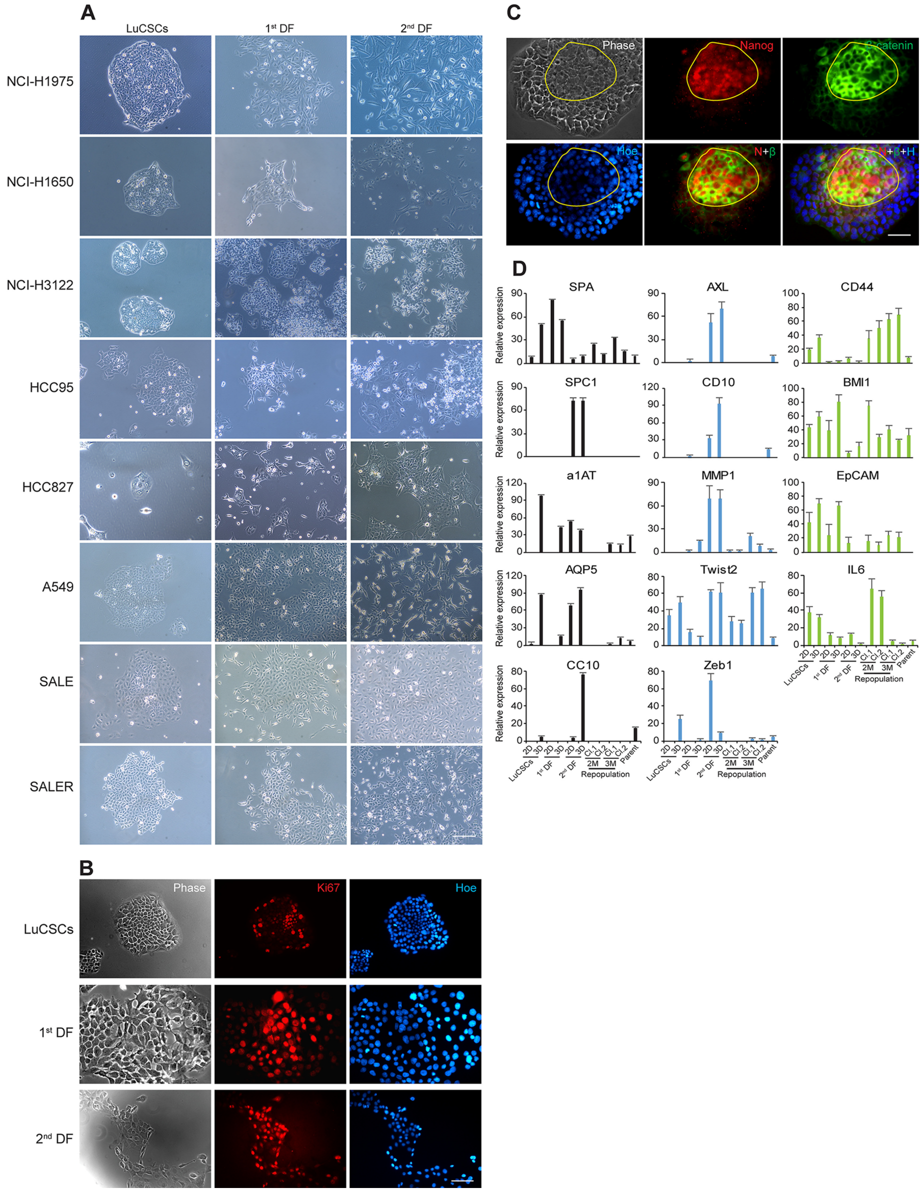

Supplementary Figure 1: Hierarchical heterogeneity of NSCLC cell lines at low cell density conditions. (A) Morphology of distinct cell clones revealed in 6 NSCLC cell lines (NCI-H1975, NCI-H1650, NCI-H3122, NCI-HCC95, HCC827 and A549), one normal airway epithelial cell line (SALE) and SALE transformed by KRAS mutant form (SALER). Scale bar: 10 µm. **(B)** Cell clones were double-labeled with primary antibody against the proliferation-associated nuclear antigen Ki67 (red) and the nuclear stain Hoechst 33342 (blue). Scale bars: 10 µm. **(C)** The initiation of LuCSC differentiation. Inner yellow line separates undifferentiated, epithelial LuCSCs from EMT-like cells on the periphery of the colony. LuCSCs were dormant and with lesser Hoechst 33342 staining. While cells in the center of the colony had strong staining for Nanog (red) and ß-catenin (green), the staining was dramatically reduced in peripheral cells. Scale bar: 20 µm. **(D)** Histogram quantification dialogs of genes evaluated in Figure 1H showing the band intensity.

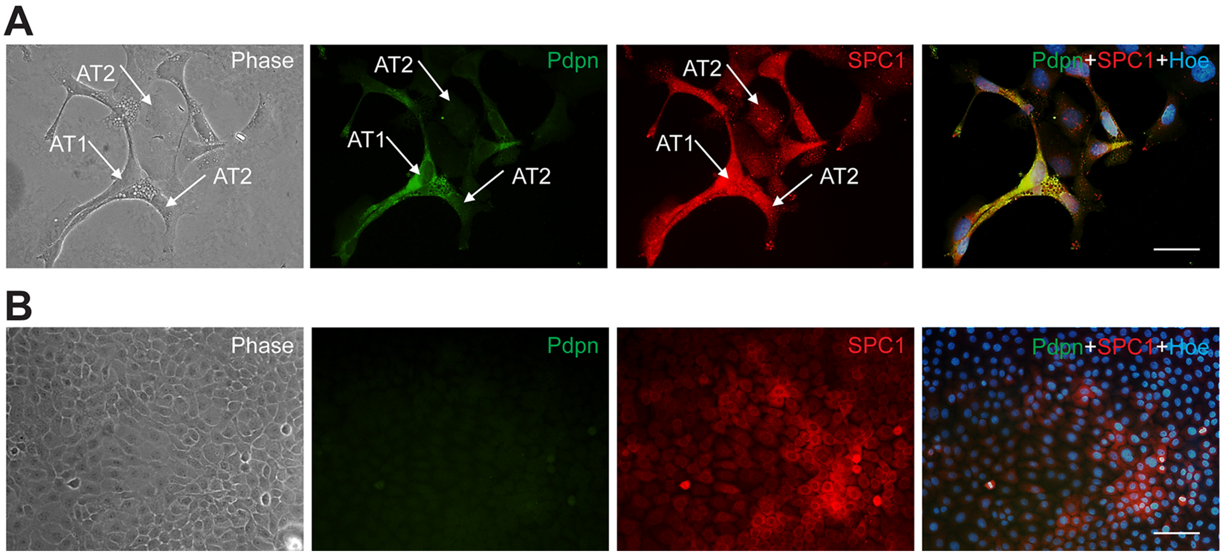

Supplementary Figure 2: Immunofluorescence characterization of 2nd DF cells. 2nd DF cells were stained with Pdpn (green) and SPC1 (red) antibodies at low **(A)** and high **(B)** density. Hoechst 33342 (blue) was used for nuclear staining. Scale bar: 20 µm.

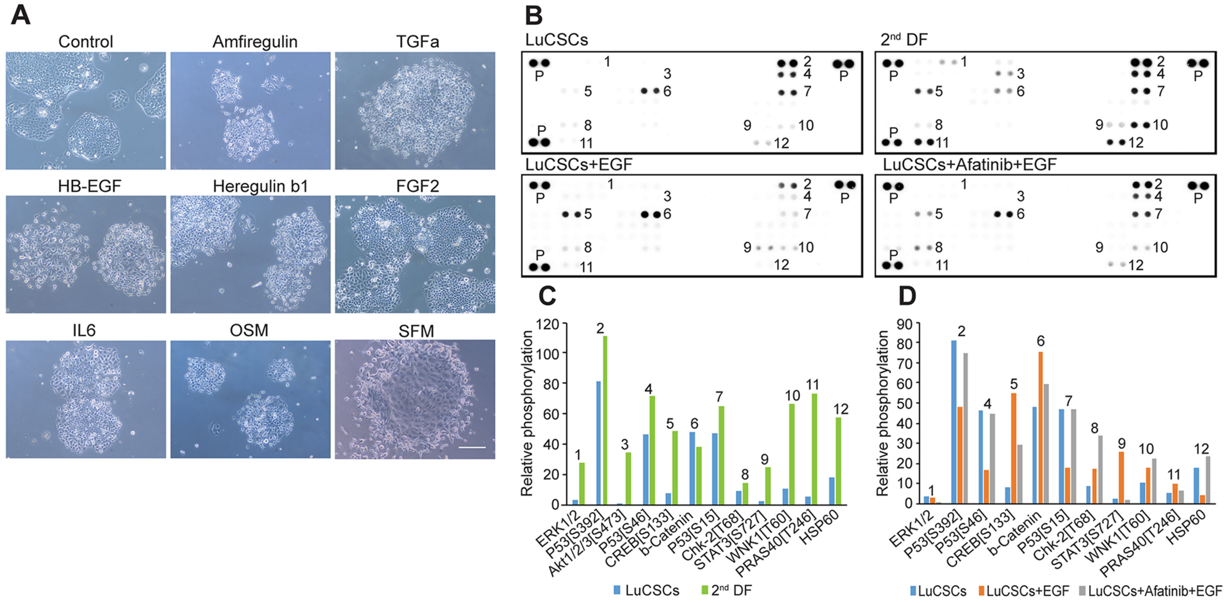

Supplementary Figure 3: Representative images of LuCSC differentiation, towards a mesenchymal phenotype, after various ligand stimulation. (A) Images were taken after 24hrs of stimulation with EGF family ligands: Amfiregulin (50 ng/ml), TGFα (5 ng/ml), HB-EGF (5 ng/ml), Heregulin b1 (50 ng/ml); FGF family: FGF2 (50 ng/ml); and IL-6 family of cytokines: IL6 (50 ng/ml), OSM (50 ng/ml) and serum free medium (SFM). **(B)** Whole-cell lysates from LuCSCs, 2nd DF cells, and EGF stimulated LuCSCs with/without Afatinib pretreatment were collected for human phospho MAPK kinase antibody array analysis. Each membrane contains kinase specific (number indicated) and positive control (P). **(C** and **D)** Relative phosphorylation of spots was quantified by normalizing pixel density of the positive control to 100. Each bar is represented as mean of duplicate spots.

Supplementary Table 1: Primers for conventional PCR

Gene	Sequence
SPA-F	5′- TTGGGAGCCTGAAAAGAAGGA -3′
SPA-R	5′- GGCTTGGAGCTCCTCATCTA -3′
SPC1-F	5′- TGGTCCTCATCGTCGTGGTGATTG -3′
SPC1-R	5′- CCTGCAGAGAGCATTCCATCTGGAAG -3′
α1AT-F	5′- TGACACTCACGATGAAATCCTGGAG -3′
α1AT-R	5′- CCTTGAGTACCCTTCTCCACGTAATC -3′
AQP5-F	5′- ACTGGGTTTTCTGGGTAGGG -3′
AQP5-R	5′- GTGGTCAGCTCCATGGTCTT -3′
CC10-F	5′- CACCCTGGTCACACTGGCTC -3′
CC10-R	5′- GGAGGGTGTCCACCAGCTTC -3′
AXL-F	5′- TTTCTCCTGCGAAGCCCATA -3′
AXL-R	5′- ATGCCACGCGGATGTGATAA -3′
CD10-F	5′- GTAAGCAGCCTCAGCCGAAC -3′
CD10-R	5′- AACAATGTCATCAGGATAGCC -3′
MMP1-F	5′- CGACTCTAGAAACACAAGAGCAAGA -3′
MMP1-R	5′- AAGGTTAGCTTACTGTCACACGCTT -3′
Twist2-F	5′- CGGGCGCCATGGAGGAGGGC -3′
Twist2-R	5′- CTAGTGGGAGGCGGACATGG -3′
Zeb1-F	5′- GTGGCCCATTACAGGCAACCAGT -3′
Zeb1-R	5′- GCTAGGCTGCTCAAGACTGTAGT -3′
CD44-F	5′- GATCCACCCCAACTCCATCTGTGC -3′
CD44-R	5′- AACTGCAAGAATCAAAGCCAAGGC -3′
BMI1-F	5′- GATGAATTCGTCACTGTGAATAACGATTT -3′
BMI1-R	5′- TCTAGATCTACAATCATTTCTGAATGCAT -3′
EpCAM-F	5′- AGCGAGTGAGAACCTACTGGA -3′
EpCAM-R	5′- GATGTCTTCGTCCCACGCA -3′
IL6-F	5′- GAGAAAGGAGACATGTAACAAGAGT -3′
IL6-R	5′- GCGCAGAATGAGATGAGTTGT -3′
EGFR-F	5′- AAGGAGCTGCCCATGAGAAA -3′
EGFR-R	5′- TGGCTTCGTCTCGGAATTTG -3′
MIG6-F	5′- GGAAGACCTACTGGAGCAGTCGCAG -3′
MIG6-R	5′- TGAGATGGACCATTTTCTGCAAAGC -3′
Tubulin-F	5′- AAGTGACAAGACCATTGGGGGAGG -3′
Tubulin-R	5′- GGGCATAGTTATTGGCAGCATC -3′

Supplementary Table 2: Primers for real-time PCR

Gene	Sequence
Nanog-F	5’-CAAAGGCAAACAACCCACTT-3′
Nanog-R	5’-TCTGCTGGAGGCTGAGGTAT-3′
Sox2-F	5’-TCCCATCACCCACAGCAAATGA-3′
Sox2-R	5’-TTTCTTGTCGGCATCGCGGTTT-3′
BMI1-F	5’-GATGAATTCGTCACTGTGAATAACGATTT-3′
BMI1-R	5’-TCTAGATCTACAATCATTTCTGAATGCAT-3′
Oct4-F	5’-ACATCAAAGCTCTGCAGAAAGAACT-3′
Oct4-R	5’-CTGAATACCTTCCCAAATAGAACCC-3′
CD133-F	5’-GCTCAGACTGGTAAATCCCC-3′
CD133-R	5’-GACTCGTTGCTGGTGAATTG-3′
Periostin-F	5’-CAGTTTTGCCCATTGACCATG-3′
Periostin-R	5’-ATAGCGCTGCGTTGTGGTG-3′
ABCG2-F	5’-AGATGGGTTTCCAAGCGTTCAT-3′
ABCG2-R	5’-CCAGTCCCAGTACGACTGTGACA-3′
ABCC1-F	5’-ATGTCACGTGGAATACCAGC-3′
ABCC1-R	5’-GAAGACTGAACTCCCTTCCT-3′
EGFR-F	5’-GTGACCGTTTGGGAGTTGATGA-3′
EGFR-R	5’-GGCTGAGGGAGGCGTTCTC-3′
Snail-F	5’-GAGGCGGTGGCAGACTAG-3′
Snail-R	5’-GACACATCGGTCAGACCAG-3′
Slug-F	5’-CATGCCTGTCATACCACAAC-3′
Slug-R	5’-GGTGTCAGATGGAGGAGGG-3′
N-Cadherin-F	5’-CTCCTATGAGTGGAACAGGAACG-3′
N-Cadherin-R	5’-TTGGATCAATGTCATAATCAAGTGCTGTA-3′
hHPRT-F	5’-GGTCCTTTTCACCAGCAAGCT-3′
hHPRT-R	5’-TGACACTGGCAAAACAATGCA-3′
